# Total Usual Nutrient Intakes of US Children (Under 48 Months): Findings from the Feeding Infants and Toddlers Study (FITS) 2016

**DOI:** 10.1093/jn/nxy042

**Published:** 2018-06-05

**Authors:** Regan L Bailey, Diane J Catellier, Shinyoung Jun, Johanna T Dwyer, Emma F Jacquier, Andrea S Anater, Alison L Eldridge

**Affiliations:** 1 Department of Nutrition Science, Purdue University, West Lafayette, IN; 2 RTI International, Research Triangle Park, NC; 3 Tufts University School of Medicine, Boston, MA; 4 Nestlé Nutrition, Florham Park, NJ; 5 Nestlé Research Center, Vers-Chez-les-Blanc, Lausanne, Switzerland

**Keywords:** nutritional epidemiology, Feeding Infants and Children Survey, FITS 2016, usual nutrient intake, dietary intake, supplement use, infants, toddlers

## Abstract

**Background:**

The US Dietary Guidelines will expand in 2020 to include infants and toddlers. Understanding current dietary intakes is critical to inform policy.

**Objective:**

The purpose of this analysis was to examine the usual total nutrient intakes from diet and supplements among US children.

**Methods:**

The Feeding Infants and Toddlers Study 2016 is a national cross-sectional study of children aged <48 mo (*n* = 3235): younger infants (birth to 5.9 mo), older infants (6–11.9 mo), toddlers (12–23.9 mo), younger preschoolers (24–36.9 mo), and older preschoolers (36–47.9 mo) based on the use of a 24-h dietary recall. A second 24-h recall was collected from a representative subsample (*n* = 799). Energy, total nutrient intake distributions, and compliance with Dietary Reference Intakes were estimated with the use of the National Cancer Institute method.

**Results:**

Dietary supplement use was 15–23% among infants and toddlers and 35–45% among preschoolers. Dietary intakes of infants were adequate, with mean intakes exceeding Adequate Intake for all nutrients except vitamins D and E. Iron intakes fell below the Estimated Average Requirement for older infants (18%). We found that 31–33% of children aged 12–47.9 mo had low percentage of energy from total fat, and >60% of children aged 24–47.9 mo exceeded the saturated fat guidelines. The likelihood of nutrient inadequacy for many nutrients was higher for toddlers: 3.2% and 2.5% greater than the Adequate Intake for fiber and potassium and 76% and 52% less than the Estimated Average Requirement for vitamins D and E, respectively. These patterns continued through older ages. Intakes exceeded the Tolerable Upper Intake Level of sodium, retinol, and zinc across most age groups.

**Conclusions:**

Dietary intakes of US infants are largely nutritionally adequate; concern exists over iron intakes in those aged 6–11.9 mo. For toddlers and preschoolers, high intake of sodium and low intakes of potassium, fiber, and vitamin D and, for preschoolers, excess saturated fat are of concern. Excess retinol, zinc, and folic acid was noted across most ages, especially among supplement users.

## Introduction

From 1992 to 2002, the National Nutrition Monitoring and Related Research Act of 1990 was instrumental in establishing an extensive framework to provide current information about the dietary exposures and nutritional status of US citizens (1). National nutrition surveys, such as the former Continuing Survey of Food Intake by Individuals and NHANES, have been used since the 1980s to inform policy and program decisions and to provide a valuable resource for research applications. Although these resources have advanced our understanding of childhood and adult nutritional status considerably, less information that is nationally representative in scope has historically been available for infants and toddlers (2). Moreover, what limited data have been collected have largely excluded both the youngest infants (i.e., those aged 0–6 mo), as well as breastfed infants, and most available data do not include nutrient exposures from dietary supplements (3), underestimating nutrient exposures from this source (4).

The Feeding Infants and Toddlers Study (FITS), a periodic national cross-sectional survey first conducted in 2002 (2) and again in 2008 (5), began extensive data collection for infants and young children that included breastfed infants and intake from dietary supplements. The 2016 FITS data come at an ideal time in the nutrition policy landscape, since the 2014 Farm Bill mandates inclusion of the so-called “B-24” age group (i.e., birth to 24 mo) in the 2020–2025 Dietary Guidelines for Americans for the first time since the Guidelines were first issued >35 y ago (6). Given the very specific nutritional needs of the B-24 age group to support rapid growth and development, understanding current trends in dietary intakes is critical to inform such policy and best tailor dietary advice for infants and children. The purpose of this study was to estimate total usual dietary intakes for energy and most nutrients among infants and children <4 y old, incorporating nutrient exposures from all sources in the FITS 2016.

## Methods

### FITS survey methods

Full details of the FITS 2016 survey selection and procedures are described elsewhere (7). Briefly, this was a nationwide, cross-sectional survey. Households were selected from 4 sampling frames. Precision requirements for subgroup analysis were achieved by partitioning the eligible population into 24 strata based on 12 age groups and participation in the Special Supplemental Nutrition Program for Women, Infants and Children (WIC). Random sampling was applied with each stratum. Sampling weights were calculated to account for the probability of household selection and then adjusted for nonresponse and incomplete coverage by calibration to reflect the US population aged birth to 47.9 mo. For this analysis, the 12 age groups were categorized as follows: younger infants (birth to 5.9 mo), older infants (6–11.9 mo), toddlers (12–23.9 mo), younger preschoolers (24–36.9 mo), and older preschoolers (36–47.9 mo). Detailed demographic information can be found in **Supplemental Table 1**.

Dietary intake data were collected from parents or caregivers by trained interviewers based on multiple-pass 24-h dietary recall and the Nutrition Data System for Research (NDSR, version 2015: University of Minnesota, Minneapolis, MN). Breast milk that was directly fed to a child was not quantified during the dietary recall. Direct breastfeeding volumes are difficult to obtain; volumes were assigned according to coding rules established for FITS 2008 (8) and based upon the extensive work of Dewey et al. and Kent et al. (9–11). The estimated breastmilk volume strategy used by FITS has been further supported in systematic review and model estimates by Da Costa et al. (12) based on stable isotope methods from 12 countries on 5 continents. The FITS methods are the standard for breastmilk assessment in the United States and are also used by the CDC (3) to estimate direct breastfeeding volumes. However, if the participant indicated that she had expressed milk, then the amount was quantified and coded as such.

A total of 3235 children <4 y old were included in the survey. A second 24-h recall was collected from 799 children to estimate within-person variance for estimating usual nutrient intakes. The study protocol and instruments were approved by the institutional review boards of RTI International, the University of Minnesota, and the Docking Institute of Public Affairs, Fort Hays State University, who also assisted with data collection in the recruitment phase.

### Statistical analysis

All statistical analyses were performed with the use of SAS (version 9, SAS Institute Inc.) and SAS-callable SUDAAN^®^ (version 11, RTI International) software. Before the diet can be characterized as at-risk for inadequacy or excess relative to Dietary Reference Intakes (DRIs), usual or long-term estimates are needed that are adjusted for random measurement error (i.e., day-to-day variation) in self-reported diet (13–15). Several statistical methods are available to adjust the 24-h recall data to remove within-person variation (i.e., random error) to better estimate usual dietary intakes (16–20). All of these methods have a similar underlying framework that a single day of intake is not representative of usual or habitual intakes and seek to remove the random error to the extent possible, and require ≥2 repeat measurements for a representative subsample of the population group of interest to allow computation of both variance components (4). For this this analysis, macros developed to implement the National Cancer Institute method (19, 20) were used to produce the mean and standard error for a given usual intake, as well as the percentiles of intake and the probabilities of meeting the Estimated Average Requirement (EAR, % <EAR) and exceeding the Adequate Intake (AI, % >AI) or Tolerable Upper Intake Level (UL, % >UL), with the use of the probability approach. For many nutrients, only an AI is available. Macronutrients were compared with the Acceptable Macronutrient Distribution Range (AMDR) for children ≥12 mo. The statistical model fit with this procedure incorporated the sampling weights and covariate adjustment for day of the week of the dietary recall (weekend/weekday), and interview sequence (first or second dietary recall). Usual intake models were based on nutrient intakes from food sources and then adjusted to account for nutrient intakes from dietary supplements as recommended (21). The dietary data are total intakes, shown for supplement users and nonusers combined. Dietary data without the inclusion of dietary supplement overestimates the prevalence of inadequacy (i.e., % <EAR) and underestimates the prevalence of potentially excessive intakes (i.e., >UL) (22–24). Estimates of nutrient intakes from foods alone by age group can be found in the **Supplemental Tables 2–6** and stratified by WIC participation in Jun et al. (25) in this supplement.

## Results

### Feeding type

The proportion of US infants fed formula is higher than that of those exclusively fed breastmilk in both infant age groups (Supplemental Table 1). Over 70% of all infants were “ever breastfed” across all age groups. By the age of 12 mo most children are not consuming breastmilk or formula.

### Dietary supplement use

Dietary supplement use varies by age group, with the highest use occurring in children aged 36–47.9 mo (**Table 1**). Across all groups, most children (>95%) are taking only one supplement product (data not shown). Among infants and children taking supplements, supplements contribute substantially towards nutrient exposures. Across all age groups, vitamin D was the most prevalent nutrient used as a dietary supplement, with a mean intake from supplements alone hovering at the AI (for children aged <12 mo) or the EAR (for children aged ≥12 mo) mark of 10 μg/d for users of dietary supplements.

**TABLE 1:** Prevalence of dietary supplement use, and amounts of selected nutrients from dietary supplements for users among US infants and children as assessed by 24-h dietary recall and stratified by age group^1^

	0–5.9 mo	6–11.9 mo	12–23.9 mo	24–35.9 mo	36–47.9 mo
Total sample size	600	902	1133	305	295
Any dietary supplement use,^2^ %	23 ± 2.3	15 ± 1.5	21 ± 1.5	35 ± 3.8	45 ± 3.9
Vitamin A,^3^ RE/d	309 ± 26 [49]	380 ± 52 [57]	357 ± 14 [193]	382 ± 19 [98]	405 ± 22 [130]
Vitamin B-6,^3^ mg/d	0.4 ± 0.03 [36]	0.72 ± 0.24 [36]	0.8 ± 0.05 [172]	1.0 ± 0.10 [94]	1.2 ± 0.07 [122]
Vitamin B-12,^3^ μg/d	2.1 ± 0.1 [19]	4.1 ± 1.3 [18]	3.1 ± 0.2 [145]	7.9 ± 4.4 [90]	3.7 ± 0.2 [122]
Niacin,^3^ mg/d	7.9 ± 0.5 [36]	9.8 ± 1.6 [34]	9.3 ± 0.5 [96]	9.2 ± 0.7 [40]	13 ± 1.5^5^ [38]
Folic acid,^3^ μg/d	NA^4^	NA^4^	155 ± 13 [102]	154 ± 14 [86]	179 ± 15 [114]
Vitamin C,^3^ mg/d	35 ± 2.0 [50]	39 ± 4.0 [54]	38 ± 4.0 [197]	49 ± 10 [106]	33 ± 3.0 [132]
Vitamin D,^3^ μg/d	10 ± 0.5 [141]	10.3 ± 0.5 [140]	9.4 ± 1.0 [223]	8.7 ± 0.7 [104]	8.5 ± 0.5 [138]
Vitamin E,^3^ mg/d	4.6 ± 0.4 [38]	9.5 ± 3.5 [41]	8.9 ± 0.6 [181]	9.8 ± 0.8 [99]	14 ± 1.0 [128]
Calcium,^3^ mg/d	NA^4^	NA^4^	119 ± 18 [34]	81 ± 8 [30]	83 ± 13 [26]
Magnesium,^3^ mg/d	NA^4^	NA^4^	17 ± 7 [23]	15 ± 5 [17]	NA^4^
Iron,^3^ mg/d	9.0 ± 1.0 [20]	12 ± 2.0 [30]	17 ± 3.0 [60]	12 ± 1.0 [28]	17 ± 3.0 [30]
Zinc,^3^ mg/d	NA^4^	NA^4^	2.7 ± 0.4 [109]	2.9 ± 0.4 [83]	3.7 ± 0.5 [112]

1 Source: Feeding Infants and Toddlers Study 2016. RE, retinol equivalents.

2 Values are mean percentages ± SEs of children taking supplements during a single 24-h recall.

3 Values are means ± SEs of nutrients from supplements among supplement users during a single 24-h recall, with the number of observations in brackets.

4 Sample size too small (*n* < 10) to make meaningful estimate.

5 Denotes the mean exceeded the Tolerable Upper Intake Level (UL). The UL established from 1998 DRI book for thiamin, riboflavin, niacin, vitamin B-6, folate, vitamin B-12, and others (26).

### Infants (birth to 12 mo)

Mean energy intakes among younger infants (i.e., those aged 0–5.9 mo) are ∼660 kcal/d, with about half coming from dietary fat (**Table 2**). For all nutrients, very high proportions of young infants exceed the AI, with vitamins D and E the only exceptions. Although the number of children taking vitamin E supplements in this age group is quite small (*n* = 38 out of 600), the average intakes from supplements among users exceeded the AI for it. Approximately 40% of young infants exceed the UL for retinol and zinc.

**TABLE 2:** Usual energy and nutrient intake distributions from foods, beverages, and dietary supplements for younger infants aged 0–5.9 mo (*n* = 600)^1^

	DRI value		Distribution of energy or nutrient intake						DRI compliance, %	
	AI^2^	UL^2^	10th	25th	50th	Mean	75th	90th	>AI	>UL
Macronutrients										
Energy, kcal/d	—	—	460	538	642	663 ± 7.1	764	894	—	—
Fat, g/d	31	—	24	29	36	37 ± 0.5	44	53	67	—
Saturated fat, g/d	—	—	9.2	12	15	16 ± 0.2	19	23	—	—
Carbohydrate, g/d	60	—	49	58	71	73 ± 0.9	85	101	71	—
Protein, g/d	—	—	7.8	10	12	13 ± 0.2	15	19	—	—
Dietary fiber, g/d	—	—	0.2	0.4	0.9	1.2 ± 0.0	1.7	2.8	—	—
Fat, % kcal	—	—	44	46	49	49 ± 0.2	52	55	—	—
Saturated fat, % kcal	—	—	17	19	21	21 ± 0.1	23	25	—	—
Carbohydrate, % kcal	—	—	36	39	43	43 ± 0.2	46	50	—	—
Protein, % kcal	—	—	6.2	6.9	7.8	7.9 ± 0.1	8.8	10	—	—
Micronutrients										
Vitamin A,^3,4^ μg RAE/d	400	600	385	474	588	604 ± 7.4	717	847	88	39
Thiamin,^3,5^ mg/d	0.2	—	0.2	0.3	0.4	0.4 ± 0.0	0.5	0.7	86	—
Riboflavin,^3,5^ mg/d	0.3	—	0.3	0.5	0.6	0.7 ± 0.0	0.9	1.2	92	—
Niacin,^3,5^ mg/d	2	—	2.3	3.2	4.5	5.2 ± 0.1	6.3	9.1	94	—
Vitamin B-6,^3,5^ mg/d	0.1	—	0.1	0.2	0.3	0.3 ± 0.0	0.4	0.6	96	—
Folate,^3,5^ μg DFE/d	65	—	60	78	105	114 ± 2.0	140	179	86	—
Vitamin B-12,^3,5^ μg/d	0.4	—	0.5	0.8	1.3	1.5 ± 0.0	2.0	2.8	92	—
Vitamin C,^3,6^ mg/d	40	—	41	54	72	76 ± 1.2	93	116	91	—
Vitamin D,^3,7^ μg/d	10	25	2.0	3.6	5.9	6.5 ± 0.2	8.7	12	17	0.04
Vitamin E,^3,6^ mg/d	4	—	1.4	2.1	3.1	3.7 ± 0.1	4.8	7.0	35	—
Vitamin K,^4^ μg/d	2	—	9	13	21	25 ± 0.7	31	45	100	—
Calcium,^7^ mg/d	200	1000	244	327	444	475 ± 8.4	589	749	95	1.9
Iron,^3,4^ mg/d	0.27	40	1.9	3.2	5.3	6.2 ± 0.2	8.2	12	100	0
Magnesium,^8^ mg/d	30	—	26	34	45	49 ± 0.8	60	76	83	—
Phosphorus,^8^ mg/d	100	—	119	164	227	246 ± 4.6	308	398	94	—
Potassium,^9^ mg/d	400	—	387	487	625	659 ± 9.7	793	978	88	—
Sodium,^9^ mg/d	120	—	107	143	195	214 ± 4.1	265	347	85	—
Zinc,^3,4^ mg/d	2	4	2.0	2.7	3.6	3.9 ± 0.1	4.8	6.1	89	41

1 Unless otherwise indicated. values are percentiles, means + SEs, or percentages of DRI compliance based on usual intakes derived from the National Cancer Institute method. Micronutrient intakes do not include dietary supplements unless indicated. Source: Feeding Infants and Toddlers Study 2016. AI, Adequate Intake; DFE, dietary folate equivalent; DRI, Dietary Reference Intake; EAR, Estimated Average Requirement; RAE, retinol activity equivalent; UL, Tolerable Upper Intake Level.

2 All macronutrient DRIs are from *Dietary reference intakes for energy, carbohydrate, fiber, fat, fatty acids, cholesterol, protein, and amino acids* (27).

3 Intake includes dietary supplements.

4 DRIs are from *Dietary reference intakes for vitamin A, vitamin K, arsenic, boron, iodine, iron, manganese, molybdenum, nickel, silicon, vanadium, and zinc* (28).

5 DRIs are from *Dietary reference intakes for thiamin, riboflavin, niacin, vitamin B6, folate, vitamin B12, pantothenic acid, biotin, and choline* (26).

6 DRIs are from *Dietary reference intakes for vitamin C, vitamin E, selenium, and carotenoids* (29).

7 DRIs are from *Dietary reference intakes for calcium and vitamin D* (30).

8 DRIs are from *Dietary reference intakes for calcium, phosphorus, magnesium, vitamin D, and fluoride* (31).

9 DRIs are from *Dietary reference intakes for water, potassium, sodium, chloride, and sulfate* (32).

For older infants (aged 6–11.9 mo), there is an established EAR for iron and zinc. About 20% of older infants are at risk for iron inadequacy, whereas <5% are at risk for zinc inadequacy (**Table 3**). However, large proportions of infants exceed the UL for zinc, and because there is virtually no use of supplements containing zinc in this age group (Table 1), the excess is coming exclusively from foods and beverages. In contrast, although about one-third of older infants exceed the UL for retinol, the sources are both diet and dietary supplements, with the average supplement dose providing about half the UL among users in this age group.

**TABLE 3:** Usual energy and nutrient intake distributions from foods, beverages, and dietary supplements for older infants aged 6–11.9 mo (*n* = 901)^1^

	DRI value			Distribution of energy or nutrient intake						DRI compliance (%)		
	EAR	AI	UL	10th	25th	50th	Mean	75th	90th	<EAR	>AI	>UL
Macronutrients^2^												
Energy, kcal/d	—	—	—	592	693	824	852 ± 7.4	982	1146	—	—	—
Fat, g/d	—	30	—	24	30	37	38 ± 0.4	46	55	—	74	—
Saturated fat, g/d	—	—	—	9	11	15	15 ± 0.2	19	23	—	—	—
Carbohydrate, g/d	—	95	—	75	89	107	111 ± 1.0	129	152	—	67	—
Protein, g/d	—	—	—	13	16	20	21 ± 0.2	24	30	—	—	—
Dietary fiber, g/d	—	—	—	2.7	4.0	5.9	6.4 ± 0.1	8.2	11	—	—	—
Fat, % kcal	—	—	—	34	37	40	40 ± 0.2	43	46	—	—	—
Saturated fat, % kcal	—	—	—	12	14	16	16 ± 0.1	18	20	—	—	—
Carbohydrate, % kcal	—	—	—	44	47	51	51 ± 0.2	54	57	—	—	—
Protein, % kcal	—	—	—	7.4	8.3	9.4	9.5 ± 0.1	11	12	—	—	—
Micronutrients												
Vitamin A,^3,4,5^ μg RAE/d	—	500	600	531	640	776	794 ± 7.2	929	1081	—	93	32
Thiamin,^3,6^ mg/d	—	0.3	—	0.4	0.5	0.6	0.7 ± 0.01	0.9	1.1	—	95	—
Riboflavin,^3,6^ mg/d	—	0.4	—	0.5	0.7	0.9	1.0 ± 0.01	1.3	1.6	—	96	—
Niacin,^3,6^ mg/d	—	4	—	4.7	6.2	8.2	8.6 ± 0.1	11	13	—	95	—
Vitamin B-6,^3,6^ mg/d	—	0.3	—	0.4	0.5	0.7	0.7 ± 0.0	0.9	1.1	—	96	—
Folate,^3,6,7^ μg DFE/d	—	80	—	112	145	190	204 ± 2.7	248	313	—	98	—
Vitamin B-12,^3,6^ μg/d	—	0.5	—	0.8	1.3	2.0	2.2 ± 0.05	2.8	3.8	—	97	—
Vitamin C,^3,8^ mg/d	—	50	—	54	70	90	95 ± 1.2	115	142	—	93	—
Vitamin D,^3,9^ μg/d	—	10	38	2.5	4.4	6.9	7.4 ± 0.2	10	13	—	24	0
Vitamin E,^3,8^ mg/d	—	5	—	2.4	3.4	5.1	5.9 ± 0.1	7.4	10	—	51	—
Vitamin K,^4^ μg/d	—	2.5	—	19	28	42	49 ± 1.0	63	88	—	100	—
Calcium,^9^ mg/d	—	260	1500	343	450	597	635 ± 8.5	780	975	—	97	0.5
Iron,^3,4^ mg/d	6.9	—	40	5.5	8.0	12	13 ± 0.2	16	21	18	—	0.04
Magnesium,^10^ mg/d	—	75	—	57	72	93	99 ± 1.2	120	149	—	72	—
Phosphorus,^10^ mg/d	—	275	—	220	290	387	412 ± 5.6	507	636	—	79	—
Potassium,^11^ mg/d	—	700	—	691	855	1074	1125 ± 13	1342	1626	—	89	—
Sodium,^11^ mg/d	—	370	—	207	273	367	400 ± 6.0	492	634	—	49	—
Zinc,^3,4^ mg/d	2.5	—	5	3.2	4.2	5.5	5.8 ± 0.1	7.0	8.7	3.5	—	59

1 Unless otherwise indicated. values are percentiles, means ± SEs, or percentages of DRI compliance based on usual intakes derived from the National Cancer Institute method. Micronutrient intakes do not include dietary supplements unless indicated. Source: Feeding Infants and Toddlers Study 2016. AI, Adequate Intake; DFE, dietary folate equivalent; DRI, Dietary Reference Intake; EAR, Estimated Average Requirement; RAE, retinol activity equivalent; UL, Tolerable Upper Intake Level.

2 All macronutrient DRIs are from *Dietary reference intakes for energy, carbohydrate, fiber, fat, fatty acids, cholesterol, protein, and amino acids* (27).

3 Intake includes dietary supplements.

4 DRIs are from *Dietary reference intakes for vitamin A, vitamin K, arsenic, boron, iodine, iron, manganese, molybdenum, nickel, silicon, vanadium, and zinc* (28).

5 The vitamin A UL is for retinol only.

6 DRIs are from *Dietary reference intakes for thiamin, riboflavin, niacin, vitamin B6, folate, vitamin B12, pantothenic acid, biotin, and choline* (26).

7 The folate UL is established for folic acid.

8 DRIs are from *Dietary reference intakes for vitamin C, vitamin E, selenium, and carotenoids* (29).

9 DRIs are from *Dietary reference intakes for calcium and vitamin D* (30).

10 DRIs are from *Dietary reference intakes for calcium, phosphorus, magnesium, vitamin D, and fluoride* (31).

11 DRIs are from *Dietary reference intakes for water, potassium, sodium, chloride, and sulfate* (32).

### Toddlers (12–23.9 mo)

Mean energy intakes for toddlers (12–23.9 mo) are 1170 kcal/d (**Table 4**). The percentage of energy contributed by fat is less than the AMDR for ∼30% of toddlers, whereas very few exceed the AMDR. Most toddlers have carbohydrate energy (87%) and protein energy (92%) within the AMDRs, respectively. More than half of toddlers have intakes of vitamin D and vitamin E that put them at risk of inadequacy as assessed by comparison to the EAR. In contrast, there is a very low prevalence of inadequacy (<3%) for vitamin A, all B vitamins, vitamin C, magnesium, and phosphorus, and only slightly higher estimates of inadequacy for calcium (9%) and iron (7%). Although the prevalence of exceeding the AI for fiber and potassium is also very low, many toddlers exceed the UL for sodium (39%), retinol (26%), and zinc (43%).

**TABLE 4:** Usual energy and nutrient intake distributions from foods, beverages, and dietary supplements for toddlers aged 12–23.9 mo (*n* = 1133)^1^

	DRI value			Distribution of energy or nutrient intake						DRI compliance (%)		
	AMDR/EAR^2^	AI	UL	10th	25th	50th	Mean	75th	90th	<AMDR/<EAR^2^	>AI	>AMDR/>UL^2^
Macronutrients^3^												
Energy, kcal/d	—	—	—	813	950	1131	1169 ± 9	1346	1573	—	—	—
Fat, g/d	—	—	—	28	34	42	44 ± 0.4	52	62	—	—	—
Saturated fat, g/d	—	—	—	10	13	17	18 ± 0.2	21	26	—	—	—
Carbohydrate, g/d	100	—	—	104	122	147	152 ± 1.2	177	207	7.9	—	—
Protein, g/d	—	—	—	29	35	44	46 ± 0.4	54	65	—	—	—
Dietary fiber, g/d	—	19	—	5.0	6.9	9.4	10.0 ± 0.1	12	16	—	3.2	—
Fat, % kcal	30–40	—	—	27	30	33	33 ± 0.1	36	39	28	—	7
Saturated fat, % kcal	—	—	—	9.4	11	13	13 ± 0.1	15	17	—	—	—
Carbohydrate, % kcal	45–65	—	—	44	47	51	51 ± 0.2	55	58	13	—	0
Protein, % kcal	5–20	—	—	13	14	16	16 ± 0.1	18	20	0	—	8
Micronutrients												
Vitamin A,^4,5,6^ μg RAE/d	210	—	600	371	458	569	587 ± 5.3	696	825	0	—	26
Thiamin,^4,7^ mg/d	0.4	—	—	0.6	0.8	1.0	1.1 ± 0.01	1.3	1.6	1.2	—	—
Riboflavin,^4,7^ mg/d	0.4	—	—	0.9	1.2	1.6	1.6 ± 0.02	2.0	2.4	0.1	—	—
Niacin,^4,7^ mg/d	5	—	—	6.8	8.7	11	12 ± 0.1	15	19	2.5	—	—
Vitamin B-6,^4,7^ mg/d	0.4	—	—	0.7	0.9	1.1	1.2 ± 0.01	1.4	1.7	0.6	—	—
Folate,^4,7,8^ μg DFE/d	120	—	300	173	222	290	308 ± 3.5	374	465	1.6	—	1.1
Vitamin B-12,^4,7^ μg/d	0.7	—	—	1.8	2.6	3.6	3.8 ± 0.05	4.8	6.0	0.3	—	—
Vitamin C,^4,9^ mg/d	13	—	400	36	47	62	66 ± 0.8	81	101	0	—	0
Vitamin D,^4,10^ μg/d	10	—	63	2.6	4.3	6.7	7.4 ± 0.2	10	13	76	—	0
Vitamin E,^4,9^ mg/d	5	—	200	2.1	3.1	4.8	6.4 ± 0.2	7.7	12	52	—	0
Vitamin K,^5^ μg/d	—	30	—	15	22	34	40 ± 0.8	51	73	—	58	—
Calcium,^10^ mg/d	500	—	2500	510	653	848	894 ± 9.9	1084	1337	9	—	0.05
Iron,^4,5^ mg/d	3	—	40	3.5	5.5	8.3	10 ± 0.2	12	17	7	—	0
Magnesium,^11^ mg/d	65	—	65	103	128	163	171 ± 1.8	205	249	0.5	—	—
Phosphorus,^11^ mg/d	380	—	3000	523	656	833	872 ± 8.9	1046	1270	1.9	—	0.003
Potassium,^12^ mg/d	—	3000	—	1102	1343	1666	1738 ± 16	2055	2468	—	2.5	—
Sodium,^12^ mg/d	—	1000	1500	805	1029	1347	1448 ± 17	1756	2217	—	77	39
Zinc,^4,5^ mg/d	2.5	—	7	3.9	5.0	6.5	6.8 ± 0.1	8.3	10	1.3	—	43

1 Unless otherwise indicated. values are percentiles, means ± SEs, or percentages of DRI compliance based on usual intakes derived from the National Cancer Institute method. Micronutrient intakes do not include dietary supplements unless indicated. Source: Feeding Infants and Toddlers Study 2016. AI, Adequate Intake; AMDR, Acceptable Macronutrient Distribution Range; DFE, dietary folate equivalent; DRI, Dietary Reference Intake; EAR, Estimated Average Requirement; RAE, retinol activity equivalent; UL, Tolerable Upper Intake Level.

2 Values are AMDRs, %<AMDRs, and %>AMDRs for macronutrients and EARs, %<EARs, and %>ULs for micronutrients, as appropriate.

3 All macronutrient DRIs are from *Dietary reference intakes for energy, carbohydrate, fiber, fat, fatty acids, cholesterol, protein, and amino acids* (27).

4 Intake includes dietary supplements.

5 DRIs are from *Dietary reference intakes for vitamin A, vitamin K, arsenic, boron, iodine, iron, manganese, molybdenum, nickel, silicon, vanadium, and zinc* (28).

6 The vitamin A UL is for retinol only.

7 DRIs are from *Dietary reference intakes for thiamin, riboflavin, niacin, vitamin B6, folate, vitamin B12, pantothenic acid, biotin, and choline* (26).

8 The folate UL is established for folic acid.

9 DRIs are from *Dietary reference intakes for vitamin C, vitamin E, selenium, and carotenoids* (29).

10 DRIs are from *Dietary reference intakes for calcium and vitamin D* (30).

11 DRIs are from *Dietary reference intakes for calcium, phosphorus, magnesium, vitamin D, and fluoride* (31).

12 DRIs are from *Dietary reference intakes for water, potassium, sodium, chloride, and sulfate* (32).

### Preschoolers (24–47.9 mo)

Mean energy intake for younger preschoolers (24–35.9 mo) is 1379 kcal (**Table 5**). Although almost all younger preschoolers had energy intakes within the AMDRs from carbohydrate (92%) and protein (94%), more than one-third had total energy from fat less than the AMDR (39%), but also exceeded the percentage of energy contribution recommendations for saturated fat (68%). A considerable proportion of younger preschoolers have at-risk intakes of vitamin D (80%), even with the use of dietary supplements. The prevalence of at-risk intakes is very low (<2%) for all B-vitamins, vitamin C, magnesium, and phosphorus, and it is only slightly higher for vitamin A (4%), calcium (6%), and iron (4%). The prevalence of exceeding the AI is very low for fiber (9%) and potassium (6%). However, almost two-thirds of younger preschoolers exceed the AI for vitamin K, and many exceed the UL for sodium (70%), retinol (46%), zinc (56%), and folic acid (∼6%).

**TABLE 5:** Usual energy and nutrient intake distributions from foods, beverages, and dietary supplements for younger preschoolers aged 24–35.9 mo (*n* = 305)^1^

	DRI value			Distribution of energy or nutrient intake						DRI compliance (%)		
	AMDR/EAR^2^	AI	UL	10th	25th	50th	Mean	75th	90th	<AMDR/<EAR^2^	**>**AI	**>**AMDR/>UL^2^
Macronutrients^3^												
Energy, kcal/d	—	—	—	963	1121	1337	1379 ± 21	1587	1858	—	—	—
Fat, g/d	—	—	—	32	38	47	49 ± 0.9	58	69	—	—	—
Saturated fat, g/d	—	—	—	11	13	17	18 ± 0.4	21	26	—	—	—
Carbohydrate, g/d	100	—	—	130	152	183	188 ± 2.9	218	256	1.3	—	—
Protein, g/d	—	—	—	34	41	50	52 ± 0.9	62	74	—	—	—
Dietary fiber, g/d	—	19	—	6.6	8.8	12	12 ± 0.3	15	19	—	9.1	—
Fat, % kcal	30–40	—	—	25	28	31	31 ± 0.3	35	38	39	—	3
Saturated fat, % kcal	<10	—	—	7.8	9.4	11	11 ± 0.2	13	15	—	—	68
Carbohydrate, % kcal	45–65	—	—	47	50	53	53 ± 0.3	57	60	5.9	—	2
Protein, % kcal	5–20	—	—	12	13	15	15 ± 0.2	17	19	0	—	6
Micronutrients												
Vitamin A,^4,5,6^ μg RAE/d	210	—	600	385	471	586	603 ± 10	714	845	4.2	—	46
Thiamin,^4,7^ mg/d	0.4	—	—	0.7	0.9	1.2	1.3 ± 0.03	1.5	1.9	0.3	—	—
Riboflavin,^4,7^ mg/d	0.4	—	—	1.0	1.3	1.7	1.8 ± 0.04	2.2	2.7	0.04	—	—
Niacin,^4,7^ mg/d	5	—	—	8.2	10	14	14 ± 0.3	17	22	0.7	—	—
Vitamin B-6,^4,7^ mg/d	0.4	—	—	0.9	1.1	1.4	1.4 ± 0.03	1.7	2.1	0.1	—	—
Folate,^4,7,8^ μg DFE/d	120	—	300	219	284	376	394 ± 8.5	482	592	0.3	—	5.7
Vitamin B-12,^4,7^ μg/d	0.7	—	—	2.2	3.1	4.5	5.5 ± 0.2	8.1	10	0.1	—	—
Vitamin C,^4,9^ mg/d	13	—	400	45	58	76	80 ± 1.7	96	119	0	—	0
Vitamin D,^4,10^ μg/d	10	—	63	2.2	3.8	6.1	6.8 ± 0.4	9.2	12	80	—	0
Vitamin E,^4,9^ mg/d	5	—	200	2.9	4.3	6.9	7.3 ± 0.2	10	12	32	—	0
Vitamin K,^5^ μg/d	—	30	—	20	29	45	52 ± 1.9	66	94	—	74	—
Calcium,^10^ mg/d	500	—	2500	554	705	915	961 ± 20	1164	1432	6.4	—	0.08
Iron, ^4,5^ mg/d	3	—	40	4	6	10	11 ± 0.4	14	20	4.1	—	0
Magnesium,^11^ mg/d	65	—	65	121	149	189	198 ± 3.8	235	287	0.4	—	—
Phosphorus,^11^ mg/d	380	—	3000	601	744	943	981 ± 19	1172	1419	0.7	—	0.004
Potassium,^12^ mg/d	—	3000	—	1251	1514	1877	1952 ± 35	2300	2760	—	6	—
Sodium,^12^ mg/d	—	1000	1500	1122	1418	1852	1978 ± 45	2390	3009	—	94	70
Zinc,^4,5^ mg/d	2.5	—	7	4.6	5.8	7.4	7.7 ± 0.1	9.2	11	0.4	—	56

1 Unless otherwise indicated. values are percentiles, means ± SEs, or percentages of DRI compliance based on usual intakes derived from the National Cancer Institute method. Micronutrient intakes do not include dietary supplements unless indicated. Source: Feeding Infants and Toddlers Study 2016. AI, Adequate Intake; AMDR, Acceptable Macronutrient Distribution Range; DFE, dietary folate equivalent; DRI, Dietary Reference Intake; EAR, Estimated Average Requirement; RAE, retinol activity equivalent; UL, Tolerable Upper Intake Level.

2 Values are AMDRs, %<AMDRs, and %>AMDRs for macronutrients and EARs, %<EARs, and %>ULs for micronutrients, as appropriate.

3 All macronutrient DRIs are from *Dietary reference intakes for energy, carbohydrate, fiber, fat, fatty acids, cholesterol, protein, and amino acids* (27).

4 Intake includes dietary supplements.

5 DRIs are from *Dietary reference intakes for vitamin A, vitamin K, arsenic, boron, iodine, iron, manganese, molybdenum, nickel, silicon, vanadium, and zinc* (28).

6 The vitamin A UL is for retinol only.

7 DRIs are from *Dietary reference intakes for thiamin, riboflavin, niacin, vitamin B6, folate, vitamin B12, pantothenic acid, biotin, and choline* (26).

8 The folate UL is established for folic acid.

9 DRIs are from *Dietary reference intakes for vitamin C, vitamin E, selenium, and carotenoids* (29).

10 DRIs are from *Dietary reference intakes for calcium and vitamin D* (30).

11 DRIs are from *Dietary reference intakes for calcium, phosphorus, magnesium, vitamin D, and fluoride* (31).

12 DRIs are from *Dietary reference intakes for water, potassium, sodium, chloride, and sulfate* (32).

Mean energy intake for older preschoolers (36–47.9 mo) was 1415 kcal (**Table 6**). Very similar themes for energy intakes from macronutrients and nutrient intakes emerge for 3-y-old children as were evident in younger preschoolers (Table 5). About 40% of older preschoolers have low energy intakes from total fats paired with high energy intakes from saturated fats. Most do not meet the vitamin D recommendations and about one-third do not meet the vitamin E recommendations. Similarly, their intakes of fiber and potassium are low compared with recommendations, although their intakes of most other nutrients were largely adequate. Older preschoolers continue to consume excessive amounts of retinol (49%), folic acid (12%), sodium (75%), and zinc (69%).

**TABLE 6:** Usual energy and nutrient intake distributions from foods, beverages, and dietary supplements for older preschoolers aged 36–47.9 mo (*n* = 295)^1^

	DRI value			Distribution of energy or nutrient intake						DRI compliance (%)		
	AMDR/EAR^2^	AI	UL	10th	25th	50th	Mean	75th	90th	<AMDR/<EAR^2^	**>**AI	**>**AMDR/>UL^2^
Macronutrients^3^												
Energy, kcal/d	—	—	—	986	1151	1370	1415 ± 22	1631	1904	—	—	—
Fat, g/d	—	—	—	32	39	48	50 ± 0.9	59	70	—	—	—
Saturated fat, g/d	—	—	—	11	13	17	18 ± 0.4	22	26	—	—	—
Carbohydrate, g/d	100	—	—	132	155	186	192 ± 3.0	222	260	1.1	—	—
Protein, g/d	—	—	—	34	41	51	54 ± 1.0	63	76	—	—	—
Dietary fiber, g/d	—	19	—	6.3	8.4	11	12 ± 0.3	15	18	—	7.5	—
Fat, % kcal	30–40	—	—	25	28	31	31 ± 0.3	35	37	41	—	3
Saturated fat, % kcal	<10	—	—	7.5	9.1	11	11 ± 0.2	13	15	—	—	63
Carbohydrate, % kcal	45–65	—	—	47	50	53	53 ± 0.3	57	60	5.5	—	2
Protein, % kcal	5–20	—	—	12	13	15	15 ± 0.2	17	19	0	—	6
Micronutrients												
Vitamin A,^4,5,6^ μg RAE/d	210	—	600	363	448	558	575 ± 10	684	810	0.8	—	49
Thiamin,^4,7^ mg/d	0.4	—	—	0.8	1.0	1.3	1.5 ± 0.04	1.7	2.5	0.2	—	—
Riboflavin,^4,7^ mg/d	0.4	—	—	1.0	1.3	1.7	1.9 ± 0.05	2.3	3.1	0.1	—	—
Niacin,^4,7^ mg/d	5	—	—	9	12	16	17 ± 0.4	21	28	0.3	—	—
Vitamin B-6,^4,7^ mg/d	0.4	—	—	0.9	1.2	1.7	2.9 ± 0.1	5.1	5.6	0.1	—	—
Folate,^4,7,8^ μg DFE/d	120	—	300	248	321	422	440 ± 9.4	537	654	0.2	—	12.3
Vitamin B-12,^4,7^ μg/d	0.7	—	—	2	3	5	8 ± 0.4	15	17	0.2	—	—
Vitamin C,^4,7^ mg/d	13	—	400	41	57	90	94 ± 2.5	127	152	0	—	0
Vitamin D,^4,10^ μg/d	10	—	63	2.4	4.1	6.6	7.1 ± 0.5	9.4	13	79	—	0
Vitamin E,^4,9^ mg/d	5	—	200	2.9	4.5	8.5	28 ± 1.6	58	61	30	—	0
Vitamin K,^5^ μg/d	—	30	—	18	26	40	47 ± 1.7	60	85	—	68	—
Calcium,^10^ mg/d	500	—	2500	510	652	845	892 ± 19	1083	1332	9.2	—	0.0
Iron,^4,5^ mg/d	3	—	40	4.6	6.9	10	13 ± 0.5	15	23	3.0	—	0
Magnesium,^11^ mg/d	65	—	65	118	146	184	193 ± 3.8	231	280	0.6	—	—
Phosphorus,^11^ mg/d	380	—	3000	600	747	943	983 ± 19	1177	1420	0.8	—	0.0
Potassium,^12^ mg/d	—	3000	—	1161	1412	1751	1824 ± 33	2157	2584	—	3.7	—
Sodium,^12^ mg/d	—	1000	1500	1185	1503	1955	2091 ± 48	2530	3169	—	96	75
Zinc,^4,5^ mg/d	2.5	—	7	5.1	6.5	8.4	8.7 ± 0.2	11	13	0.3	—	69

1 Unless otherwise indicated. values are percentiles, means + SEs, or percentages of DRI compliance based on usual intakes derived from the National Cancer Institute method. Micronutrient intakes do not include dietary supplements unless indicated. Source: Feeding Infants and Toddlers Study 2016. AI, Adequate Intake; AMDR, Acceptable Macronutrient Distribution Range; DFE, dietary folate equivalent; DRI, Dietary Reference Intake; EAR, Estimated Average Requirement; RAE, retinol activity equivalent; UL, Tolerable Upper Intake Level.

2 Values are AMDRs, %<AMDRs, and %>AMDRs for macronutrients and EARs, %<EARs, and %>ULs for micronutrients, as appropriate.

3 All macronutrient DRIs are from *Dietary reference intakes for energy, carbohydrate, fiber, fat, fatty acids, cholesterol, protein, and amino acids* (27).

4 Intake includes dietary supplements.

5 DRIs are from *Dietary reference intakes for vitamin A, vitamin K, arsenic, boron, iodine, iron, manganese, molybdenum, nickel, silicon, vanadium, and zinc* (28).

6 The vitamin A UL is for retinol only.

7 DRIs are from *Dietary reference intakes for thiamin, riboflavin, niacin, vitamin B6, folate, vitamin B12, pantothenic acid, biotin, and choline* (26).

8 The folate UL is established for folic acid.

9 DRIs are from *Dietary reference intakes for vitamin C, vitamin E, selenium, and carotenoids* (29).

10 DRIs are from *Dietary reference intakes for calcium and vitamin D* (30).

11 DRIs are from *Dietary reference intakes for calcium, phosphorus, magnesium, vitamin D, and fluoride* (31).

12 DRIs are from *Dietary reference intakes for water, potassium, sodium, chloride, and sulfate* (32).

## Discussion

In FITS 2016, some consistent themes emerged that were also apparent in earlier iterations of the FITS (33, 34) as well as recent NHANES data (3). Total usual intakes of infants were by and large nutritionally adequate, but once family or table foods start to predominate the diet at ∼12 mo of age (35), suboptimal intake of some nutrients appeared. This was particularly notable for high-sodium and low-potassium intakes (33, 34). Current recommendations suggest that intakes of potassium should be higher than those of sodium, and yet the reverse is apparent among these children starting at ∼12 mo of age (32). Across all age groups, excessive intakes of vitamin A (retinol), zinc, and folic acid (in some age groups) were also evident, often paired with low intakes of fiber, vitamin D, and vitamin E. Low intakes of iron and calcium were also of concern, but only in some age groups (33, 34).

At ≥12 mo of age, the percentage of total energy intake contributed from dietary fat decreased and the percentage of contribution from protein increased and remained relatively constant until 48 mo of age, which was congruous with American Academy of Pediatrics recommendations (36). However, substantial proportions of toddlers and children aged 2–3 y were below the AMDR for total fat. Based on the high proportion of children exceeding the energy requirement for saturated fat, the types of fats consumed by children aged >12 mo are not the optimal ones, potentially putting them at risk for suboptimal intakes of essential fatty acids.

The FITS data represent usual intake exposures that are adjusted for measurement error to the extent possible. This is the first report of usual total nutrient intakes in the 0- to 5.9-mo age group; FITS 2008 was only able to estimate 1-d means (33). Usual intake means that single-day estimates of intake are adjusted for random measurement error; this adjustment is particularly important when looking at the tails of the distributions, or the prevalence of individuals at risk for inadequacy or excess (4). We also present total usual intakes, inclusive of dietary supplements; these estimates are particularly salient because of the high proportion of supplement use within certain age groups among infants, toddlers, and children aged 2–3 y. Previous FITS studies have shown dietary intakes for users and nonusers combined (33, 34) and separated (37). In FITS 2016, we presented dietary supplement users combined with nonusers to permit estimation of national prevalence trends for inadequacy and excess. However, data presented this way tend to underestimate nutritional exposures for supplement users and overestimate nutritional exposures for nonusers (22–24). Most of the dietary supplements used in this age group do not contain fiber, sodium, macronutrients, or potassium. In infants, most supplement use was confined to vitamin D, usually in droplet form (data not shown). Once children can chew and swallow, multiple nutrient supplements (i.e., gummies and multivitamins) start to contribute more towards nutrient exposures for many nutrients. Nonetheless, the nutrient estimates from foods alone (Supplemental data), do not substantially differ from total intakes (inclusive of supplements) with the exception of vitamins D and E in terms of meeting the AI and the EAR for all age groups. The prevalence of exceeding the UL increased substantially when dietary supplements are considered for vitamin A (≥12 mo of age) and for zinc (≥36 mo of age). Given that there is very little nutrient inadequacy of vitamin A and zinc in these age groups, dietary supplements are not necessarily essential for meeting recommendations and manufacturers could potentially reformulate the amount of these nutrients provided in their products.

Our data suggest that among children aged ≥12 mo it is difficult to obtain the recommended vitamin D intakes without the use of supplements. FITS 2016 data presented elsewhere in this supplement also suggest that children <4 y old who are participating in WIC have more optimal vitamin D intakes than those who are not (25). It should be noted that estimates of vitamin D inadequacy from the diet are often higher than that of estimates from serum 25 (OH) vitamin D (which includes the contribution of sunlight exposure), but little is known about the relation between dietary intakes and biomarkers of vitamin D exposure in infants (38).

A new finding from the FITS 2016 data is that the use of dietary supplements has increased among infants and toddlers, with a substantial change the 0- to 5.9-mo age group from 9% reported in FITS 2008 to 23% in FITS 2016 (37). This increased use (predominantly from the use of vitamin D–containing supplements) dovetails nicely with the increase in exclusive breastfeeding rates and concomitant American Academy of Pediatrics recommendations for the use of vitamin D supplements in infants.

We observed a higher prevalence of iron inadequacy among older infants (18%), which parallels the finding that infant cereal consumption has decreased in this age group and infant meats are not consumed by many (39). The prevalence of iron inadequacy reported here is also higher than was estimated for infants in NHANES 2009–2012 (10% <EAR), which did not account for dietary supplement use as we did in this study (3). Moreover, the prevalence of iron inadequacy was higher in FITS 2016 than in FITS 2008 and 2002 for all children aged ≥12 mo (33).

The majority of the available DRIs were set between 1997 and 2005, and only 2 nutrient DRIs have been updated since then: calcium and vitamin D in 2011 (30). Little experimental research is available on nutrient requirements of infants and very young children. Across all age groups, and especially <12 mo of age, the availability of EARs is limited for appropriately evaluating intakes for adequacy, especially for infants <12 mo for whom there are only AIs. AIs are derived from published intake distributions or estimated mean intake supplied by human milk for healthy, exclusively breastfed infants (with the exception of vitamin D) (4). An AI is assumed to exceed the RDA for a nutrient, if one could be established. Thus, in applying the AI, the proportion of a group that exceeds the AI should reflect those who have adequate intakes, but there is no scientific basis to state that the proportion of intakes lower than the AI is an estimate of the prevalence of inadequacy. Exceeding the AI essentially means that the average intakes of infants from birth to 12 mo is quite high. Since most dietary supplements in this age group are confined to vitamin D, nutrients are obtained through foods and beverages (i.e., formula, breastmilk, and juice). Less than two-thirds to half of infants and children were meeting the recommendations for intakes of vitamin E. The ULs for young infants and children have been criticized as having been established with too little available data and are considered to be too low for many nutrients (40). Indeed, for zinc, ∼40% of US infants and children exceed the UL from diet alone. Although total vitamin A intakes exceeded the UL in a large proportion of infants and toddlers, it should also be noted that substantial numbers exceeded the UL for vitamin A from foods alone. Thus, the ULs for vitamin A and zinc should be considered for re-evaluation, since so many children exceed them, whereas the EAR for vitamin E deserves review since so many children fail to meet it without any documented adverse health effects. The formulations of dietary supplements targeted to infants and children need to be optimized both to more closely match their nutritional needs, and to reduce the risk of excessive exposures based on the current ULs.

The strengths of this study include new population-based data on total usual nutrient intakes from foods and dietary supplements among children aged <4 y, with emphasis on very young infants and toddlers. The major limitation of this cross-sectional survey is that diet was measured by self-report from parents and caregivers. FITS and other large studies are useful for examining cross-sectional trends in dietary intakes over time to develop hypotheses on total intakes and specific foods and beverages that may increase risks of pediatric overweight and obesity. However, studies that collect longitudinal data, especially with the inclusion of biomarker and anthropometric measurements, are also needed to fill these gaps and to best inform research, clinical practice, and policy.

In conclusion, dietary intakes of US infants (<12 mo of age) are largely adequate, exceeding the AIs for all nutrients except vitamins D and E, but the likelihood of inadequacy increases in toddlers and preschoolers. Iron remains a nutrient of concern in older infants, with almost 1 in 5 below the EAR. Many of the nutrients of concern identified in the existing Dietary Guidelines for Americans, including low fiber, vitamin D, and potassium intakes and high sodium intakes, were also observed in this data for children aged >12 mo. Additional nutrients of concern at all ages in this analysis are excessive retinol and zinc (and to a lesser extent folic acid) intakes, especially among children taking dietary supplements that contain these nutrients. Children of all ages in FITS 2016 had satisfactory intakes of B-vitamins, vitamins C and K, and most minerals except those that were previously noted. Concern for the balance of the types of fats consumed by children aged >12 mo is warranted, with concomitant low overall energy intakes from total fat and high percentages of energy intakes from saturated fat.

## Supplementary Material

Supplement Tables

## Notes

Published in a supplement to *The Journal of Nutrition*. Select contents of this supplement were presented at the Experimental Biology 2017 conference in a session titled, “Informing B-24 Dietary Guidelines: Findings from the New Feeding Infants and Toddlers Study 2016,” held in Chicago, IL, April 22, 2017. Supplement Coordinators were William Dietz, Milken School of Public Health at the George Washington University and Andrea S. Anater, RTI International. The FITS research was funded by the Nestle Research Centre (Lausanne, Switzerland) through a contract with RTI International and its subcontractor, the University of Minnesota. Dr. Dietz is a compensated consultant to RTI on this project. The article contents are the responsibility of the authors and do not necessarily represent the opinions or recommendations of Nestle. Publication costs for this supplement were defrayed in part by the payment of page charges. This publication must therefore be hereby marked “advertisement” in accordance with 18 USC section 1734 solely to indicate this fact. The opinions expressed in this publication are those of the authors and are not attributable to the sponsors or the publisher, Editor, or Editorial Board of *The Journal of Nutrition*.

Supported by Nestlé Research Center, Vers-Chez-les-Blanc, Route du Jorat 57, Case Postale 44, 1000 Lausanne-26, Switzerland.

Author disclosures: EFJ and ALE are employees of the Nestlé Research Center (funding source). RLB was a scientific consultant of RTI International for the FITS 2016 project. JTD is a member of the Scientific Advisory Boards of Conagra Foods and McCormick Spice, has served as a consultant for Gerber/Nestle, and has received partial travel expenses to moderate a symposium at the International Congress of Nutrition in Buenos Aires in 2017 from the International Alliance of Dietary Supplement Associations. She holds stock in several food and drug companies. DJC, SJ, and ASA, Dr. Dwyer is a member of the Scientific Advisory Boards of Conagra Foods and McCormick Spice, has served as a consultant for Gerber/Nestle, and has received partial travel expenses to moderate a symposium at the International Congress of Nutrition in Buenos Aires Argentina in 2017 from the International Alliance of Dietary Supplement Associations. She holds stock in several food and drug companies. Bailey is a consultant to RTI International.

Supplemental Tables 1–6 are available from the “Supplementary data” link in the online posting of the article and from the same link in the online table of contents at https://academic.oup.com/jn/.

Abbreviations used:

AI: Adequate Intake
AMDR: Acceptable Macronutrient Distribution Range
DRI: Dietary Reference Intake
EAR: Estimated Average Requirement
FITS: Feeding Infants and Toddlers Study
UL: Tolerable Upper Intake Level
WIC: Special Supplemental Nutrition Program for Women, Infants and Children
