# Supplementary material for: Total Usual Nutrient Intakes of US Children (Under 48 Months): Findings from the Feeding Infants and Toddlers Study (FITS) 2016
Source: J Nutr. 2018 Jun 5;148(Suppl 3):1557S–1566S. doi: 10.1093/jn/nxy042 (PMC6126633; doi:10.1093/jn/nxy042)
Supplement: Supplement Tables [file nxy042_supplement_tables.docx]

**Supplemental Table 1. Proportion (%) of FITS 2016 sample that identify with selected characteristics by age group^1^**

| **Variable** | **All ages** | **0-5.9 mo** | **6-11.9 mo** | **12-23.9 mo** | **24-35.9 mo** | **36-47.9 mo** |
| --- | --- | --- | --- | --- | --- | --- |
| **n** | 3,235 | 600 | 902 | 1,133 | 305 | 295 |
| **Child ever breastfed** | 82±0.7 | 86±1.4 | 85±1.2 | 81±1.2 | 77±2.4 | 73±2.6 |
| **Breast Milk/Formula Category**^2^ |  |  |  |  |  |  |
| Breast milk only | 12±0.7 | 34±2.5 | 26±1.7 | 12 ±1.2 | 6±1.7 | 2±1.1 |
| Formula only | 14±0.7 | 43±2.8 | 55±2.1 | 8 ±1.2 | 0 | 0 |
| Both (breast milk & formula) | 5±0.4 | 22±2.3 | 15±1.5 | 1 ±0.5 | 0 | 0 |
| Neither (no breast milk or formula) | 69 ±1.1 | 1±0.6 | 4±0.8 | 79±1.6 | 93±1.7 | 97±1.2 |
| **Child's Race/Ethnicity** |  | |  | |  | |
| Hispanic | 15±0.6 | 16±1.5 | 14±1.2 | 14±1.0 | 14±2.0 | 14±2.0 |
| Non-Hispanic White | 67±0.8 | 65±2.0 | 68±1.6 | 68±1.4 | 64±2.7 | 66±2.8 |
| Non-Hispanic Black | 14±0.6 | 13±1.4 | 13±1.1 | 13±1.0 | 20±2.3 | 17±2.2 |
| Non-Hispanic Other | 4±0.4 | 6±1.0 | 5±0.7 | 4±0.6 | 3±1.0 | 3±1.0 |
| **Respondent’s Education** |  | |  | |  | |
| < High school | 4±0.4 | 4±0.9 | 4±0.6 | 3±0.5 | 5±1.4 | 6±1.4 |
| High school or equivalent | 19±0.7 | 19±1.6 | 18±1.3 | 18±1.2 | 21±2.3 | 25±2.5 |
| Some college/postsecondary | 23±0.7 | 27±1.8 | 24±1.4 | 23±1.3 | 18±2.2 | 17±2.2 |
| Completed college | 38±0.9 | 37±2.0 | 38±1.6 | 41±1.5 | 36±2.8 | 36±2.8 |
| Some graduate work/degree | 15±0.6 | 13±1.4 | 15±1.2 | 15±1.1 | 20±2.3 | 16±2.1 |
| **Relationship to Child** |  | |  | |  | |
| Biological/adoptive mother | 80±0.7 | 82±1.6 | 82±1.3 | 79±1.2 | 75±2.5 | 74±2.6 |
| Biological/adoptive father | 11±0.5 | 12±1.3 | 11±1.0 | 11±0.9 | 8±1.6 | 10±1.8 |
| Other | 10±0.5 | 6±1.0 | 7±0.9 | 10±0.9 | 17±2.1 | 16±2.1 |
| **Current Marital Status** |  | |  | |  | |
| Married | 70±0.8 | 71±1.9 | 72±1.5 | 71±1.4 | 66±2.7 | 64±2.8 |
| Separated or divorced | 6±0.4 | 4.9±0.9 | 3.5±0.6 | 6.5±0.7 | 9±1.7 | 8±1.6 |
| Widowed | 1±0.2 | 0.7±0.3 | 0.1±0.1 | 0.5±0.2 | 2±0.9 | 3±1.0 |
| Never married | 11±0.6 | 9±1.2 | 11±1.1 | 11±0.9 | 13±1.9 | 14±2.0 |
| Living with partner | 12±0.6 | 15±1.4 | 14±1.14 | 11±0.9 | 10±1.8 | 11±1.8 |
| **Household Income** |  | |  | |  | |
| Less than $10,000 | 8±0.5 | 9±1.2 | 8±0.9 | 8±0.8 | 11±1.8 | 7±1.5 |
| $10,000 to $19,999 | 9±0.5 | 10±1.2 | 9±1.0 | 8±0.8 | 11±1.8 | 13±1.9 |
| $20,000 to $34,999 | 18±0.7 | 18±1.6 | 21±1.4 | 18±1.1 | 14±2.0 | 14±2.0 |
| $35,000 to $49,999 | 17±0.7 | 17±1.5 | 18±1.3 | 17±1.1 | 12±1.9 | 15±2.1 |
| $50,000 to $74,999 | 20±0.7 | 20±1.6 | 19±1.3 | 22±1.2 | 19±2.3 | 16±2.1 |
| $75,000 to $99,999 | 14±0.6 | 14±1.4 | 13±1.1 | 13±1.0 | 15±2.1 | 17±2.2 |
| $100,000 to $149,000 | 10±0.5 | 8±1.1 | 9±1.0 | 11±0.9 | 14±2.0 | 12±1.9 |
| $150,000 or more | 4±0.3 | 4±0.8 | 3±0.6 | 4±0.6 | 4±1.2 | 7±1.5 |
| **Other** |  | |  | |  | |
| Low birth weight (< 2.5 kg) | 9±0.5 | 6±1.0 | 9±1.0 | 8±0.8 | 10±1.7 | 14±2.0 |
| Mother works | 47±1.0 | 37±2.2 | 43±1.8 | 49±1.7 | 57±3.3 | 60±3.3 |
| Father works | 84±2.0 | 91±3.4 | 78±4.2 | 89±2.8 | 80±8.0 | 73±8.1 |
| Any SNAP participation | 26±0.8 | 24±1.8 | 26±1.5 | 26±1.3 | 30±2.6 | 29±2.6 |
| Attends daycare^3^ | 36±0.8 | 24±1.7 | 31±1.5 | 36±1.4 | 52±2.9 | 64±2.8 |

SNAP = Supplemental Nutrition Assistance Program

^1^ Values are percentage of respondents ± SE. Columns do not always add to 100% due to missing data, rounding, and overlapping response options.

^2^ These categories refer only to consumption of breast milk and formula; children may also consume other foods, including cow’s milk, in any of these categories. Data are from the feeding practices questionnaire and represent general feeding practices, not just day of recall. A child in the *breast milk only* category is still breastfeeding at least occasionally and is not receiving formula at all, but may well be eating other foods.

^3^ Includes preschool, daycare, and care by a baby sitter or any other person who is not the parent.

**Supplemental Table 2. Usual nutrient intake distributions from foods and beverages for younger infants aged 0 to 5.9 mo (n=600), Feeding Infants and Toddlers Study 2016**

|  | **DRI Value** | | **Distribution of Energy or Nutrient Intake^1^** | | | | | | **DRI Compliance (%)** | |
| --- | --- | --- | --- | --- | --- | --- | --- | --- | --- | --- |
| **Micronutrients** | **AI** | **UL** | **10th** | **25th** | **50th** | **Mean** | **75th** | **90th** | **>AI** | **>UL** |
| Vitamin A (μg RAE/d) | 400 | 600 | 385 | 474 | 588 | 604±7.4 | 717 | 847 | 88 | 35 |
| Thiamin (mg/d) | 0.2 | – | 0.2 | 0.2 | 0.4 | 0.4±0.01 | 0.5 | 0.6 | 85 | ‒ |
| Riboflavin (mg/d) | 0.3 | – | 0.3 | 0.4 | 0.6 | 0.7±0.01 | 0.8 | 1.1 | 91 | – |
| Niacin (mg/d) | 2 | – | 2.3 | 3.1 | 4.4 | 4.7±0.09 | 5.9 | 7.6 | 94 | – |
| Vitamin B-6 (mg/d) | 0.1 | – | 0.1 | 0.2 | 0.3 | 0.3±0.01 | 0.4 | 0.5 | 96 | – |
| Folate (μg DFE/d) | 65 | – | 60 | 78 | 105 | 114±2.0 | 140 | 179 | 86 | – |
| Vitamin B-12 (μg/d) | 0.4 | – | 0.4 | 0.8 | 1.2 | 1.4±0.04 | 1.9 | 2.6 | 92 | – |
| Vitamin C (mg/d) | 40 | – | 41 | 53 | 69 | 73±1.2 | 89 | 111 | 90 | – |
| Vitamin D (μg/d) | 10 | 25 | 1.4 | 2.7 | 4.6 | 5.3±0.14 | 7.1 | 9.9 | 9.8 | 0.01 |
| Vitamin E (mg/d) | 4 | – | 1.3 | 2.0 | 3.0 | 3.5±0.08 | 4.4 | 6.2 | 31 | – |
| Calcium (mg/d) | 200 | 1,000 | 244 | 327 | 444 | 475±8.4 | 589 | 749 | 95 | 1.9 |
| Iron (mg/d) | 0.27 | 40 | 1.8 | 3.2 | 5.2 | 5.9±0.2 | 7.9 | 11 | 100 | 0 |
| Zinc (mg/d) | 2 | 4 | 2.0 | 2.7 | 3.6 | 3.8±0.07 | 4.8 | 6.0 | 89 | 40 |

AI, Adequate Intake; DFE, dietary folate equivalent; DRI, Dietary Reference Intake; RAE, retinol activity equivalent; UL, Tolerable Upper Intake Level.

^1^ Values are percentiles, mean±SE, and percentage of DRI compliance based on usual intakes derived from the National Cancer Institute method. Intakes do not include dietary supplements

**Supplemental Table 3. Usual nutrient intake distributions from foods and beverages for older infants aged 6-11.9 mo (n=901), Feeding Infants and Toddlers Study 2016**

|  | **DRI Value** | | | **Distribution of Energy or Nutrient Intake^1^** | | | | | | **DRI Compliance (%)** | | |
| --- | --- | --- | --- | --- | --- | --- | --- | --- | --- | --- | --- | --- |
| **Micronutrients** | [**EAR**](http://www.journals.elsevierhealth.com/periodicals/yjada/article/S0002-8223%2810%2901480-X/fulltext#tblfn15) | **AI** | **UL** | **10th** | **25th** | **50th** | **Mean** | **75th** | **90th** | **<EAR** | **>AI** | **>UL** |
| Vitamin A (μg RAE/d) | – | 500 | 600 | 531 | 640 | 776 | 794±7.2 | 929 | 1081 | – | 93 | 30 |
| Thiamin (mg/d) | – | 0.3 | – | 0.4 | 0.5 | 0.6 | 0.7±0.009 | 0.8 | 1.0 | – | 95 | – |
| Riboflavin (mg/d) | – | 0.4 | – | 0.5 | 0.7 | 0.9 | 1.0±0.01 | 1.2 | 1.5 | – | 96 | – |
| Niacin (mg/d) | – | 4 | – | 4.7 | 6.1 | 8.1 | 8.5±0.1 | 10 | 13 | – | 95 | – |
| Vitamin B-6 (mg/d) | – | 0.3 | – | 0.4 | 0.5 | 0.7 | 0.7±0.009 | 0.9 | 1.1 | – | 96 | – |
| Folate (μg DFE/d) | – | 80 | – | 112 | 145 | 190 | 203±2.7 | 247 | 310 | – | 98 | – |
| Vitamin B-12 (μg/d) | – | 0.5 | – | 0.8 | 1.3 | 1.9 | 2.1±0.04 | 2.7 | 3.6 | – | 97 | – |
| Vitamin C (mg/d) | – | 50 | – | 54 | 69 | 89 | 93±1.1 | 113 | 139 | – | 93 | – |
| Vitamin D (μg/d) | – | 10 | 38 | 2.1 | 3.6 | 5.8 | 6.4±0.1 | 8.6 | 11.6 | – | 17 | 0 |
| Vitamin E (mg/d) | – | 5 | – | 2.4 | 3.4 | 4.9 | 5.6±0.1 | 7.1 | 9.6 | – | 49 | – |
| Calcium (mg/d) | ‒ | 260 | 1500 | 343 | 450 | 597 | 635±8.5 | 779 | 975 | ‒ | 97 | 0.5 |
| Iron (mg/d) | 6.9 | – | 40 | 5.4 | 7.9 | 11 | 12±0.2 | 16 | 20 | 18 | – | 0.04 |
| Zinc (mg/d) | 2.5 | – | 5 | 3.2 | 4.2 | 5.5 | 5.8±0.07 | 7.0 | 8.6 | 3.5 | ‒ | 59 |

AI, Adequate Intake; DFE, dietary folate equivalent; DRI, Dietary Reference Intake; RAE, retinol activity equivalent; UL, Tolerable Upper Intake Level.

^1^ Values are percentiles, mean±SE, and percentage of DRI compliance based on usual intakes derived from the National Cancer Institute method. Intakes do not include dietary supplements

**Supplemental Table 4. Usual nutrient intake distributions from foods and beverages for toddlers aged 12-23.9 mo (n=1,133) Feeding Infants and Toddlers Study 2016**

|  | **DRI Value** | | **Distribution of Energy or Nutrient Intake^1^** | | | | | | **DRI Compliance (%)** | |
| --- | --- | --- | --- | --- | --- | --- | --- | --- | --- | --- |
| **Micronutrients** | [**EAR**](http://www.journals.elsevierhealth.com/periodicals/yjada/article/S0002-8223%2810%2901480-X/fulltext#tblfn15) | **UL** | **10th** | **25th** | **50th** | **Mean** | **75th** | **90th** | **<EAR** | **>UL** |
| Vitamin A (μg RAE/d) | 210 | 600 | 371 | 458 | 569 | 587±5.3 | 696 | 825 | 0 | 15 |
| Thiamin (mg/d) | 0.4 | – | 0.6 | 0.8 | 1.0 | 1.0±0.01 | 1.2 | 1.5 | 1.4 | – |
| Riboflavin (mg/d) | 0.4 | – | 0.9 | 1.2 | 1.5 | 1.6±0.02 | 1.9 | 2.3 | 0.1 | – |
| Niacin (mg/d) | 5 | – | 6.6 | 8.4 | 11 | 11±0.1 | 14 | 17 | 2.7 | – |
| Vitamin B-6 (mg/d) | 0.4 | – | 0.7 | 0.8 | 1.1 | 1.1±0.01 | 1.3 | 1.6 | 0.7 | – |
| Folate (μg DFE/d) | 120 | 300 | 169 | 214 | 278 | 296±3.4 | 358 | 446 | 1.7 | 0.8 |
| Vitamin B-12 (μg/d) | 0.7 | – | 1.8 | 2.4 | 3.4 | 3.6±0.05 | 4.5 | 5.7 | 0.4 | – |
| Vitamin C (mg/d) | 13 | 400 | 34 | 45 | 60 | 63±0.8 | 78 | 97 | 0 | 0 |
| Vitamin D (μg/d) | 10 | 63 | 2.5 | 4.1 | 6.5 | 7.2±0.1 | 9.5 | 13 | 78 | 0 |
| Vitamin E (mg/d) | 5 | 200 | 2.0 | 2.8 | 4.2 | 4.8±0.08 | 6.1 | 8.3 | 62 | 0 |
| Calcium (mg/d) | 500 | 2500 | 509 | 652 | 846 | 892±9.9 | 1082 | 1335 | 9.3 | 0.05 |
| Iron (mg/d) | 3 | 40 | 3.4 | 5.3 | 8.0 | 8.8±0.1 | 12 | 15 | 7.0 | 0 |
| Zinc (mg/d) | 2.5 | 7 | 3.8 | 4.8 | 6.3 | 6.6±0.07 | 7.9 | 9.7 | 1.4 | 38 |

AI, Adequate Intake; DFE, dietary folate equivalent; DRI, Dietary Reference Intake; RAE, retinol activity equivalent; UL, Tolerable Upper Intake Level.

^1^ Values are percentiles, mean±SE, and percentage of DRI compliance based on usual intakes derived from the National Cancer Institute method. Intakes do not include dietary supplements

**Supplemental Table 5. Usual nutrient intake distributions from foods and beverages for younger preschoolers aged 24-35.9 mo (n=305) Feeding Infants and Toddlers Study 2016**

|  | **DRI Value** | | **Distribution of Energy or Nutrient Intake^1^** | | | | | | **DRI Compliance (%)** | |
| --- | --- | --- | --- | --- | --- | --- | --- | --- | --- | --- |
| **Micronutrients** | [**EAR**](http://www.journals.elsevierhealth.com/periodicals/yjada/article/S0002-8223%2810%2901480-X/fulltext#tblfn15) | **UL** | **10th** | **25th** | **50th** | **Mean** | **75th** | **90th** | **<EAR** | **>UL** |
| Vitamin A (μg RAE/d) | 210 | 600 | 385 | 471 | 586 | 603±10 | 714 | 845 | 4.2 | 18 |
| Thiamin (mg/d) | 0.4 | – | 0.7 | 0.9 | 1.1 | 1.2±0.02 | 1.4 | 1.7 | 0.4 | – |
| Riboflavin (mg/d) | 0.4 | – | 1.0 | 1.3 | 1.6 | 1.7±0.04 | 2.1 | 2.5 | 0.04 | – |
| Niacin (mg/d) | 5 | – | 8.0 | 10 | 13 | 13±0.3 | 16 | 20 | 0.8 | – |
| Vitamin B-6 (mg/d) | 0.4 | 30 | 0.8 | 1.0 | 1.2 | 1.3±0.02 | 1.5 | 1.8 | 0.1 | – |
| Folate (μg DFE/d) | 120 | 300 | 205 | 258 | 334 | 353±7.5 | 425 | 529 | 0.4 | 2.3 |
| Vitamin B-12 (μg/d) | 0.7 | – | 2.0 | 2.7 | 3.7 | 3.9±0.09 | 4.9 | 6.1 | 0.2 | – |
| Vitamin C (mg/d) | 13 | 400 | 42 | 53 | 70 | 74±1.6 | 91 | 113 | 0 | 0 |
| Vitamin D (μg/d) | 10 | 63 | 2.2 | 3.8 | 6.1 | 6.7±0.2 | 8.9 | 12 | 82 | 0 |
| Vitamin E (mg/d) | 5 | 200 | 2.5 | 3.6 | 5.2 | 5.9±0.2 | 7.4 | 10 | 47 | 0 |
| Calcium (mg/d) | 500 | 2,500 | 547 | 693 | 900 | 94+±20 | 1,144 | 1,411 | 6.9 | 0.07 |
| Iron (mg/d) | 3 | 40 | 4.2 | 6.2 | 9.3 | 10±0.3 | 13 | 17 | 4.1 | 0.01 |
| Zinc (mg/d) | 2.5 | 7 | 4.3 | 5.5 | 7.1 | 7.4±0.1 | 8.9 | 11 | 0.6 | 51 |

AI, Adequate Intake; DFE, dietary folate equivalent; DRI, Dietary Reference Intake; RAE, retinol activity equivalent; UL, Tolerable Upper Intake Level.

^1^ Values are percentiles, mean±SE, and percentage of DRI compliance based on usual intakes derived from the National Cancer Institute method. Intakes do not include dietary supplements

**Supplemental Table 6. Usual nutrient intake distributions from foods and beverages for older preschoolers aged 36-47.9m (n=295), Feeding Infants and Toddlers Study 2016**

|  | **DRI Value** | | **Distribution of Energy or Nutrient Intake^1^** | | | | | | **DRI Compliance (%)** | | |
| --- | --- | --- | --- | --- | --- | --- | --- | --- | --- | --- | --- |
| **Micronutrients** | [**EAR**](http://www.journals.elsevierhealth.com/periodicals/yjada/article/S0002-8223%2810%2901480-X/fulltext#tblfn15) | **UL** | **10th** | **25th** | **50th** | **Mean** | **75th** | **90th** | | **<EAR** | **>UL** |
| Vitamin A (μg RAE/d) | 210 | 600 | 363 | 448 | 558 | 575±10 | 684 | 810 | | 0.8 | 15 |
| Thiamin (mg/d) | 0.4 | – | 0.8 | 1.0 | 1.2 | 1.3±0.02 | 1.5 | 1.8 | | 0.2 | – |
| Riboflavin (mg/d) | 0.4 | – | 1.0 | 1.2 | 1.6 | 1.7±0.04 | 2.0 | 2.5 | | 0.07 | – |
| Niacin (mg/d) | 5 | – | 9.2 | 12 | 15 | 15±0.3 | 18 | 22 | | 0.3 | – |
| Vitamin B-6 (mg/d) | 0 | 30 | 0.8 | 1.0 | 1.3 | 1.3±0.02 | 1.6 | 1.9 | | 0.1 | – |
| Folate (μg DFE/d) | 120 | 300 | 223 | 280 | 361 | 382±8.2 | 460 | 569 | | 0.3 | 4.8 |
| Vitamin B-12 (μg/d) | 0.7 | – | 1.9 | 2.6 | 3.5 | 3.7±0.09 | 4.7 | 5.9 | | 0.3 | – |
| Vitamin C (mg/d) | 13 | 400 | 36 | 47 | 62 | 66±1.5 | 81 | 101 | | 0 | 0 |
| Vitamin D (μg/d) | 10 | 63 | 2.0 | 3.4 | 5.6 | 6.2±0.2 | 8.4 | 11 | | 85 | 0 |
| Vitamin E (mg/d) | 5 | 200 | 2.4 | 3.5 | 5.1 | 5.7±0.2 | 7.3 | 9.9 | | 49 | 0 |
| Calcium (mg/d) | 500 | 2,500 | 506 | 648 | 842 | 887±19 | 1078 | 1,328 | | 9.5 | 0.05 |
| Iron (mg/d) | 3 | 40 | 4.5 | 6.7 | 9.8 | 11±0.3 | 14 | 18 | | 3.2 | 0.03 |
| Zinc (mg/d) | 2.5 | 7 | 4.6 | 5.8 | 7.4 | 7.7±0.2 | 9.3 | 11 | | 0.4 | 56 |

AI, Adequate Intake; DFE, dietary folate equivalent; DRI, Dietary Reference Intake; RAE, retinol activity equivalent; UL, Tolerable Upper Intake Level.

^1^ Values are percentiles, mean±SE, and percentage of DRI compliance based on usual intakes derived from the National Cancer Institute method. Intakes do not include dietary supplements
